# Two-year outcomes of modified external suture trabeculotomy for Chinese open-angle glaucoma patients

**DOI:** 10.3389/fmed.2026.1760525

**Published:** 2026-05-11

**Authors:** Zhan Xie, Yangyang Lu, Haiyue Xie, Zhengtong Xu, Mulong Du, Ying-Ting Zhu, Ping Xie, Hong Sun

**Affiliations:** 1 Department of Ophthalmology, The First Affiliated Hospital with Nanjing Medical University, Nanjing, Jiangsu, China; 2 Nanjing Qixia District Hospital, Nanjing, China; 3 Department of Biostatistics, School of Public Health, Nanjing Medical University, Nanjing, Jiangsu, China; 4 R&D Department, BioTissue, Miami, FL, United States

**Keywords:** 2-year outcome, IOP, modified external suture trabeculotomy, open-angle glaucoma, Schlemm’s canal

## Abstract

**Background:**

This study aimed to evaluate the 2-year outcomes of modified external suture trabeculotomy in the treatment of open-angle glaucoma (OAG) among Chinese patients.

**Methods:**

A retrospective study was designed, involving 138 OAG patients (138 eyes) treated with modified external suture trabeculotomy at the First Affiliated Hospital of Nanjing Medical University from January 2020 to June 2023. The patients were followed up at 1 week, 1 month, 3 months, 6 months, 1 year, and 2 years. Surgical success was defined as intraocular pressure (IOP) ≤ 21 mmHg (1 mmHg = 0.133 kPa) under glaucoma medication (conditional success) and without any glaucoma medication (complete success). The main outcomes were measured according to IOP, number of medication regimens, surgical success rate, and complications.

**Results:**

A total of 138 OAG patients (138 eyes) were included, with a mean age of 58.57 ± 13.66 years (range, 25–86 years). The mean postoperative IOP decreased from preoperative 24.59 ± 9.82 to postoperative 15.50 ± 6.49, 15.70 ± 5.76, 14.36 ± 3.59, 14.49 ± 3.96, 14.71 ± 4.31, and 14.31 ± 3.16 mm Hg at 1 week, 1 month, 3 months, 6 months, 1 year, and 2 years, respectively. The mean numbers of medication regimens were 2 (0 ~ 4), 0 (0 ~ 3), 0 (0 ~ 3), 0 (0 ~ 3), 0 (0 ~ 2), 0 (0 ~ 3), 0 (0 ~ 3) at the seven time points, respectively. The mean postoperative IOP and the number of medication regimens at each time point were significantly lower than those before the operation (all *p* < 0.01). The 2-year total success rate was 93.3%, and the complete success rate was 82.5%. Hyphema (79.0%) and transient rise of IOP (16.7%) were the most common complications early after surgery. Descemet’s membrane detachment occurred in nine eyes (6.5%), and ciliary body detachment in four eyes (2.9%).

**Conclusion:**

Simple and cost-effective, the modified external suture trabeculotomy provides favorable 2-year outcomes in treating medically uncontrolled OAG, as shown by short surgical duration, effective IOP control, significant drug reduction, and fewer postoperative interventions.

## 1 Introduction

Glaucoma, represented by open-angle glaucoma (OAG), is the leading cause of irreversible blindness globally (1). In the condition of OAG, the juxtacanalicular tissue of the trabecular meshwork and the inner wall of Schlemm’s canal (SC) impart the greatest resistance to aqueous outflow (2). Therefore, procedures that remove, disrupt, or bypass this resistance can facilitate physiologic outflow and consequently lower IOP (2).

Many canal-oriented procedures have been developed, including circumferential trabeculotomy (3–6). Trabeculotomy can be subtyped into metal trabeculotomy (7), microcatheter-assisted trabeculotomy (MAT) (8), and suture trabeculotomy (9). In these procedures, metal probes can only open part of the trabecular meshwork in the superior temporal and superior nasal quadrants. However, both the collecting channel openings and aqueous veins are most densely distributed in the inferonasal quadrant (7). Therefore, metal probes may fail to cut through them during trabeculotomy (10, 11). In addition, when inserted into the SC, the metal probe may unintentionally form false channels in the anterior chamber angle and the suprachoroidal space (7). In MAT surgery, a luminescent microcatheter can precisely locate the SC, allowing for a 360-degree incision into its outer wall and trabecular meshwork tissue. However, its expense restricts a wider application, especially in developing countries. Furthermore, internal MAT surgery cannot be easily performed in patients with corneal edema or corneal nebula. Simpler, more economical surgical procedures are urgently needed.

To meet this need, we have designed a cost-saving external suture trabeculotomy, in which a 5–0 polypropylene suture, rather than the illuminated microcatheter, is introduced to cannulate the SC (9). This modification enables identification of the SC and circumferential trabeculotomy (9, 12). Later, we simplified this technique and validated its long-term efficacy. Inflammation is closely related to the prognosis of trabeculotomy. Hong et al. (13) have demonstrated that higher concentrations of CCL2, ICAM-1, IL-6, and CXCL10 in the aqueous humor are associated with trabeculotomy failure. Masato et al. (14) have reported that after trabeculotomy in 75 primary open-angle glaucoma (POAG) patients, peripheral anterior synechiae (PAS) occurs in 86% of the eyes, with 39% within the incision and 13% outside the incision. Based on these findings, we have further optimized our technique by omitting the preparation of the deep scleral flap, adding peripheral iridectomy, and unsuturing the conjunctival incision. These improvements are expected to alleviate the inflammatory response during surgery, reduce postoperative PAS, and increase the complete success rate of the surgery.

Herein, we evaluated the 2-year outcomes of this modified external suture trabeculotomy in a consecutive series of Chinese patients with OAG.

## 2 Methods

### 2.1 Study design and participants

Enrolled were 138 OAG patients (138 eyes) treated with modified external suture trabeculotomy in the First Affiliated Hospital of Nanjing Medical University, China, from January 2020 to June 2023. This protocol was approved by the Ethics Committee of the First Affiliated Hospital of Nanjing Medical University (2025-SR-601) and complies with the Declaration of Helsinki. Written informed consent was obtained from all patients before surgery.

### 2.2 Inclusion criteria

1. Patients had been previously diagnosed as only POAG or juvenile open-angle glaucoma (JOAG).
2. Patients showed uncontrolled OAG defined as IOP > 21 mmHg or progressive deterioration of visual field (VF) defects on maximal medications. Maximal medications referred to four types of anti-glaucoma drugs, or fewer than four types of anti-glaucoma drugs, but with intolerance.
3. Patients received successful procedures of modified external suture trabeculotomy and a follow-up ≥ 24 months.
4. If both eyes of a patient had undergone modified external suture trabeculotomy, the data of the right eye would be selected for statistical analysis.

### 2.3 Exclusion criteria

1. Secondary open-angle glaucoma (e.g., uveitic glaucoma, steroid-induced glaucoma, posttraumatic glaucoma, glaucoma due to intraocular tumors, and caused by elevated episcleral venous pressure).
2. Patients presented with severe heart and lung diseases and advanced cancer, and could not tolerate ophthalmic surgery.
3. Patients presented with a previous intraocular surgery history and penetrating ocular trauma.
4. Patients presented with ocular fundus vascular diseases, such as diabetic retinopathy, central or branch retinal vein occlusion.
5. Patients refused to sign the informed consent form and could not meet the follow-up requirements of this study.

### 2.4 Surgical procedures

**Figure 1:**
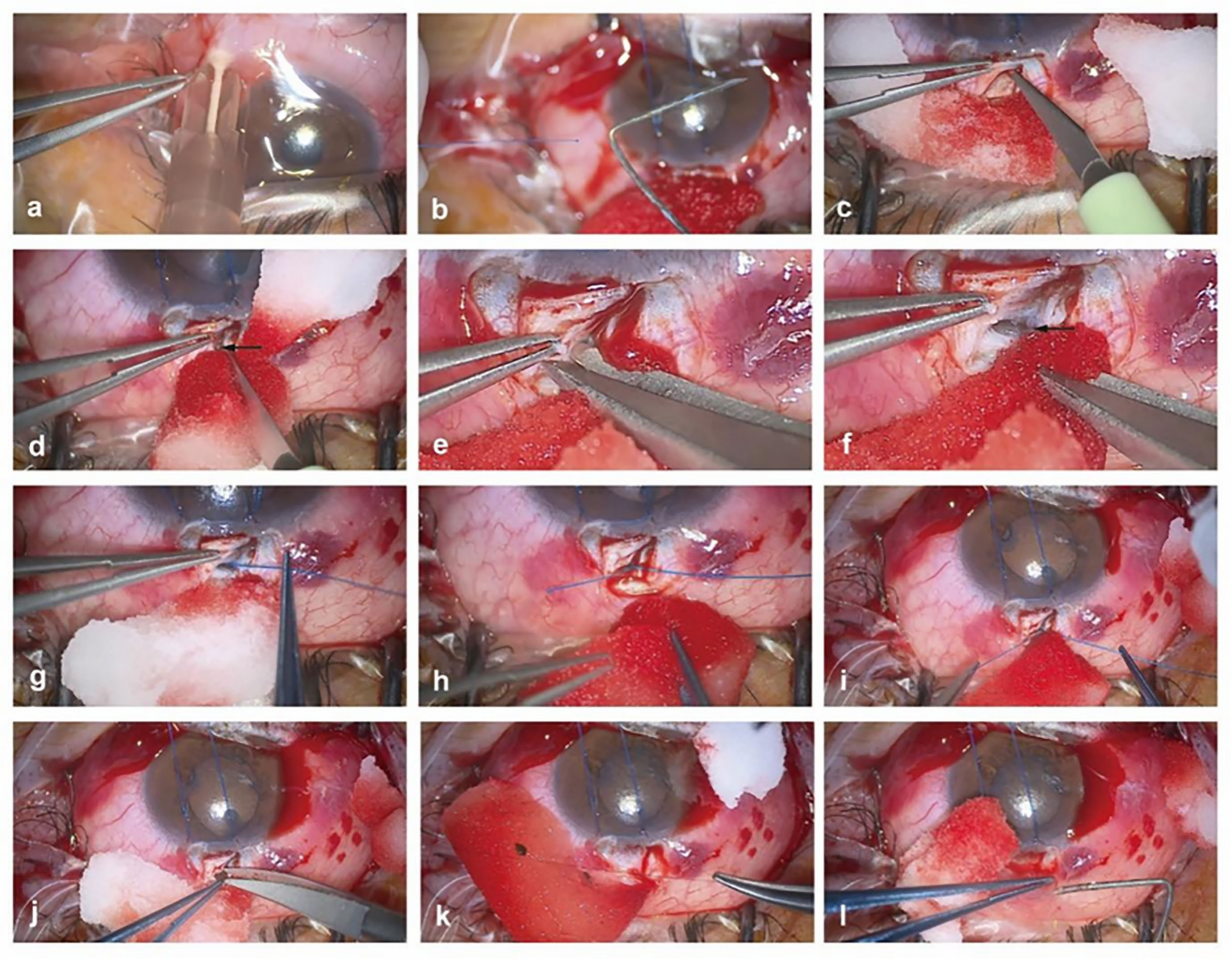
Procedures of modified external suture trabeculotomy. **(a)** Performing Subtenon’s anesthesia with 2% lidocaine+ 0.75% bupivacaine (2.5 mL, mixed in a ratio of 1:1). **(b)** Burning the end of a standard 6–0 polypropylene suture into a small ball. **(c)** Dissecting a rectangular superficial scleral flap of 4 × 4 mm and in 1/2 scleral thickness at the superior limbus into the transparent cornea. **(d)** Locating SC. Black arrow indicating the broken end of the SC. **(e)** Inserting the corneal scissors into the broken end of the SC. **(f)** Opening the outer wall of the SC. Black arrow indicating the unroofed SC. **(g)** Inserting the tip of the polypropylene suture into one ostium of the SC. **(h)** Passing the suture through nearly the entire circumference of the SC. **(i)** 360-degree resection. **(j)** Peripheral iridectomy. **(k)** Closing the scleral flap tightly with one suture of 10–0 nylon. **(l)** Injecting moderate anesthetics under the conjunctival flap without suturing the conjunctival incision.

All surgeries were performed by one designated experienced surgeon at the First Affiliated Hospital of Nanjing Medical University. Peribulbar anesthesia was achieved through injection of 2% lidocaine + 0.75% bupivacaine (2.5 mL, mixed in a ratio of 1:1) (Figure 1a). A suspension wire was made of 8–0 polypropylene suture in the corneal limbus. A fornix-based incision was made through the conjunctiva and Tenon’s capsule. After cauterization, the tip of the 6–0 polypropylene suture was mushroom-shaped with a rough and sharp margin. The tip size varied depending on cauterization energy (Figure 1b). Then, a 4 × 4 mm superficial scleral flap in 1/2 scleral thickness was sculpted (Figure 1c). The SC was located (Figure 1d), and its outer wall was opened using the corneal scissors (Figures 1e,f). After paracentesis of the anterior chamber, the tip of the polypropylene suture was inserted into one ostium of the SC (Figure 1g) and passed through the canal’s entire circumference (Figure 1h). Then, a 360-degree resection was performed (Figure 1i). Following resection of the peripheral iris at 12 o’clock (Figure 1j), the scleral flap was tightly closed with one suture of 10–0 nylon (Figure 1k). Moderate anesthetics was injected under the conjunctival flap without suturing the conjunctival incision (Figure 1l). The corneal incisions were hydrated and made watertight. Antimetabolic agents, such as mitomycin C or 5-fluorouracil, were not used intraoperatively.

### 2.5 Observational indexes

Patients were followed up at 1 week, 1 month, 3 months, 6 months, 1 year, and 2 years, with additional visits whenever necessary, for evaluating the surgical outcomes according to IOP, number of anti-glaucoma regimens, best corrected visual acuity (BCVA), results of anterior and posterior segment examination, frequency of complications, postoperative interventions, and morphology of filtering blebs.

### 2.6 Evaluation of surgical success or failure

The surgery was defined as success or failure according to the following criteria (15, 16).

(1) Complete success: post-operative IOP at 6 to 21 mmHg without anti-glaucoma medications;
(2) Conditional success: post-operative IOP at 6 to 21 mmHg with local application of anti-glaucoma medications;
(3) Total success: post-operative IOP at 6 to 21 mmHg with or without anti-glaucoma medications;
(4) Failure: post-operative IOP lower than 6 mmHg or higher than 21 mmHg after application of anti-glaucoma medications. Surgical failure also included the presence of severe complications, such as retinal detachment and endophthalmitis.

### 2.7 Statistical analysis

SPSS version 21.0 was used for statistical analyses. Continuous variables were summarized as means, standard deviations, medians, and ranges; categorical variables as frequencies and percentages. For continuous variables, *t*-test was performed for normally distributed samples, while a corresponding non-parametric test was used for non-normally distributed samples. Repeated-measures analysis of variance (ANOVA) was used to analyze the IOP changes in patients at different time points. Chi-square analysis was used to analyze the drug use before and after surgery. Kaplan–Meier analysis was performed to calculate the cumulative rate of total success in OAG patients. Statistical significance was defined as a *p*-value of <0.05.

## 3 Results

**Figure 2:**
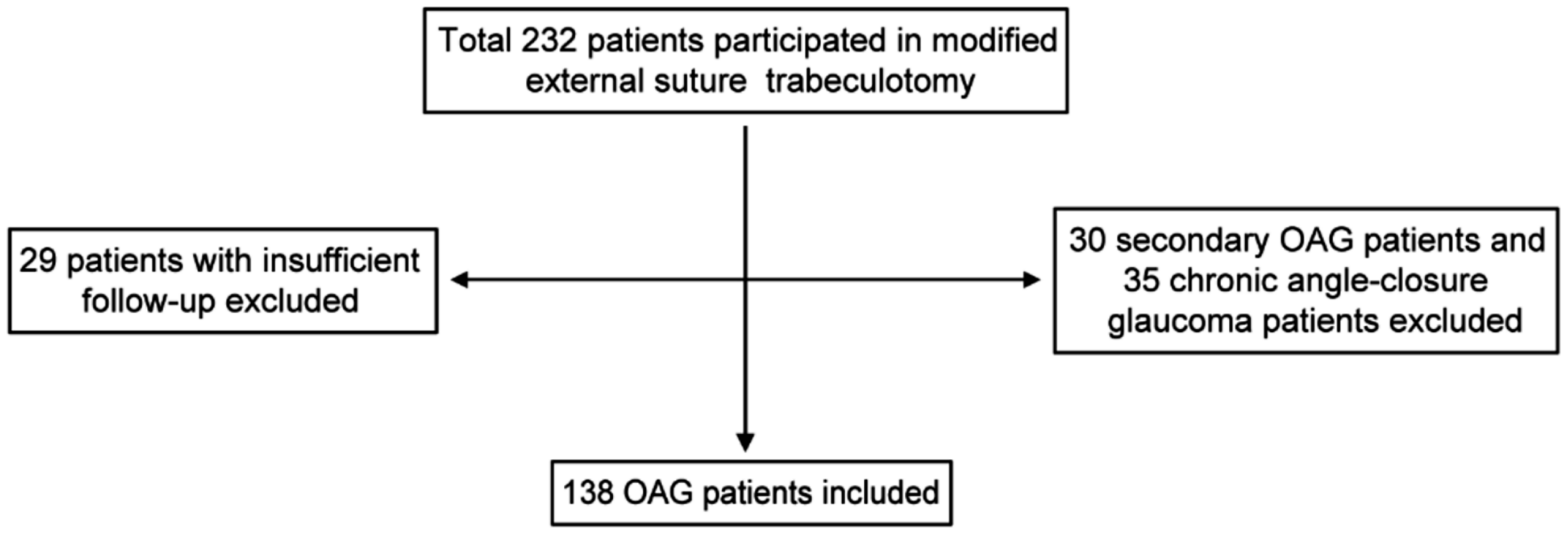
Flow diagram for the inclusion of patients for successful modified external suture trabeculotomy and follow-up from January 2020 to June 2023.

A total of 138 OAG patients (138 eyes), including 98 men, were recruited in this study (Figure 2), with a mean age of 58.57 ± 13.66 years (range 25–86 years), an average preoperative IOP of 24.59 ± 9.82 mmHg, and a median (range) number of 2 (0 ~ 4) regimens. Patient characteristics are presented in Table 1.

**Table 1:** Characteristics of patients enrolled.

Characteristics	Values
No. of eyes/patients	138/138
OD/OS	96/42
Gender (male/female)	98/40
Age	58.57 ± 13.66
Preoperative IOP (mmHg)	24.59 ± 9.82
Preoperative medications	2.01 ± 1.07
Preoperative BCVA (LogMAR)	0.44 ± 0.26
Phakic eye (*n*, %)	70 (50.7%)
Diagnosis (*n*, %)	
JOAG	17 (12.3%)
POAG	121 (87.7%)
Surgical procedure (*n*, %)	
Modified external suture trabeculotomy	70 (50.7%)
Modified external suture trabeculotomy + Phaco	68 (49.3%)

Data were expressed as mean ± SD.

IOP, intraocular pressure; SD, standard deviation; BCVA, best-corrected visual acuity; JOAG, Juvenile-onset open-angle glaucoma; POAG, primary open-angle glaucoma; Phaco, phacoemulsification.

**Figure 3:**
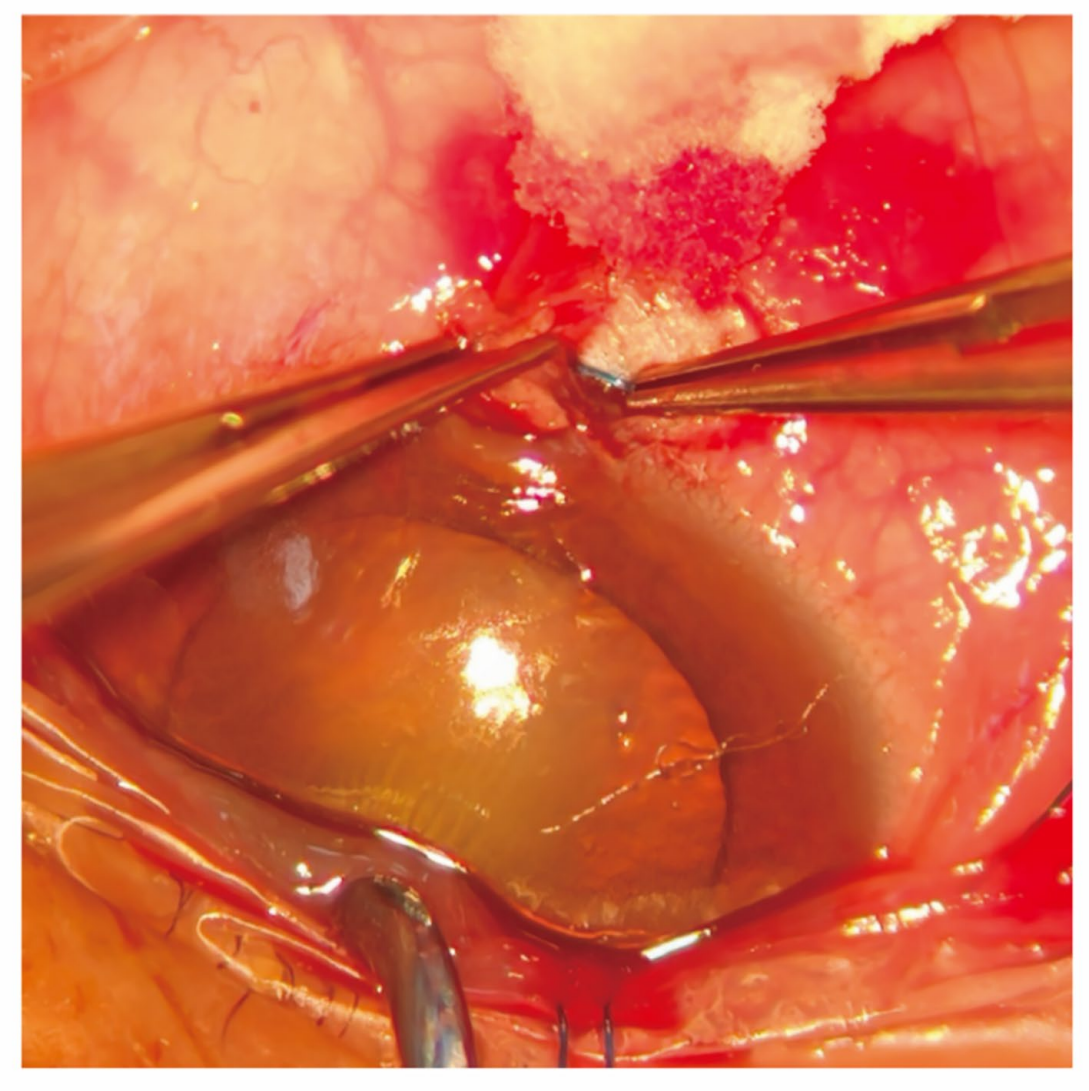
Relay catheterization in the inferior angle when the SC could not be cannulated in a patient with OAG. In this case, the 6–0 polypropylene suture reached an obstruction and became stranded. At the position (usually the lower angle of the chamber) where the suture encountered resistance, the surgeon made an incision. The conjunctiva was then incised, and the sclera was cut down over the suture tip, which was retrieved at the point of obstruction. Then, the suture was placed into one cut end of the canal and gradually advanced.

Catheterization was successful in 94.2% cases with 6–0 polypropylene suture (Table 2). In six cases, the 6–0 polypropylene suture reached an obstruction or strayed into a new direction. Relay catheterization in the inferior angle was performed when the SC could not be cannulated (Figure 3). In two cases, cannulation could not be achieved smoothly through the 6–0 suture, which was then replaced with a 5–0 polypropylene suture (Table 2). In two cases, because the passages frequently proved false during cannulation, we used a 7–0 polypropylene suture for cannulation (Table 2). In two cases, the suture could not be turned by at least 180 degrees within the canal in either clockwise or counterclockwise direction; therefore, a traditional Harms metal trabeculotomy was performed, in which the suture was placed into the presumed canal beneath the original scleral flap (Table 2).

**Table 2:** Duration of surgery and success or failure of catheterization.

Parameter	Values
Duration of surgery (min)	16.72 ± 2.98
Modified external suture trabeculotomy	14.33 ± 1.27
Modified external suture trabeculotomy + Phaco	19.18 ± 2.09
Successful catheterization	
6–0 polypropylene suture	130 (94.2%)
5–0 polypropylene suture	2 (1.4%)
7–0 polypropylene suture	2 (1.4%)
Failed catheterization	2 (1.4%)
Harms metal trabeculotomy	2 (1.4%)

Data were expressed as mean ± SD.

Phaco, phacoemulsification.

**Figure 4:**
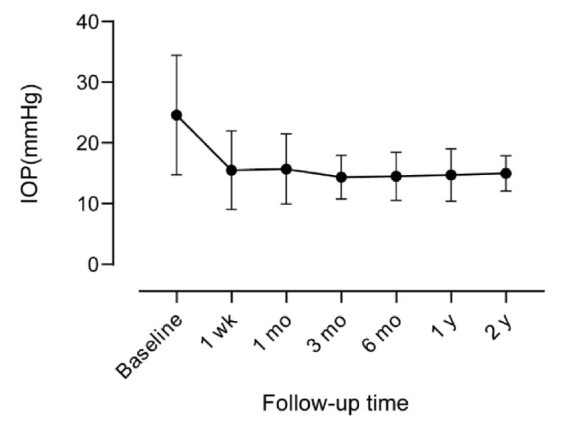
IOP values at baseline and six time points in the follow-up. IOP values were shown as mean ± SD. SD, standard deviations; IOP, intraocular pressure.

The mean IOP decreased from preoperative 24.59 ± 9.82 to postoperative 15.50 ± 6.49, 15.70 ± 5.76, 14.36 ± 3.59, 14.49 ± 3.96, 14.71 ± 4.31, and 14.31 ± 3.16 mmHg at 1 week, 1 month, 3 months, 6 months, 1 year, and 2 years, respectively. Shown by Table 3 and Figure 4, the difference between the mean IOP values at baseline and each follow-up point was statistically significant (*p* < 0.001). The mean numbers of medication regimens were 2 (0 ~ 4), 0 (0 ~ 3), 0 (0 ~ 3), 0 (0 ~ 3), 0 (0 ~ 2), 0 (0 ~ 3), 0 (0 ~ 3) at the seven time points, respectively. The difference in the number of anti-glaucoma regimens between baseline and each follow-up point was statistically significant (*p* < 0.001; Table 3). BCVA at post-operative 2 years was 0.41 ± 0.31, not significantly different from that at baseline (*p* = 0.265).

**Table 3:** IOP and medications after completing modified external suture trabeculotomy.

Follow-up time	N	IOP (mmHg)	Eyes with meds/total (%)	Number of meds regimens
Pre-operation	138	24.59 ± 9.82	127 (92.0)	2 (0 ~ 4)
1 week	138	15.50 ± 6.49	26 (18.8)	0 (0 ~ 3)
1 month	138	15.70 ± 5.76	14 (10.1)	0 (0 ~ 3)
3 months	138	14.36 ± 3.59	8 (5.8)	0 (0 ~ 3)
6 months	138	14.49 ± 3.96	10 (7.3)	0 (0 ~ 2)
1 year	138	14.71 ± 4.31	21 (15.2)	0 (0 ~ 3)
2 year	120	14.31 ± 3.16	21 (17.5)	0 (0 ~ 3)
*p*-value		<0.001^*^	<0.001^†^	

IOP data were expressed as mean ± SD, Meds data were expressed as median (range), IOP, intraocular pressure; SD, standard deviation; meds, medications.

^*^Repeated-measures analysis of variance (ANOVA) analysis.

^†^Chi-square analysis.

**Figure 5:**
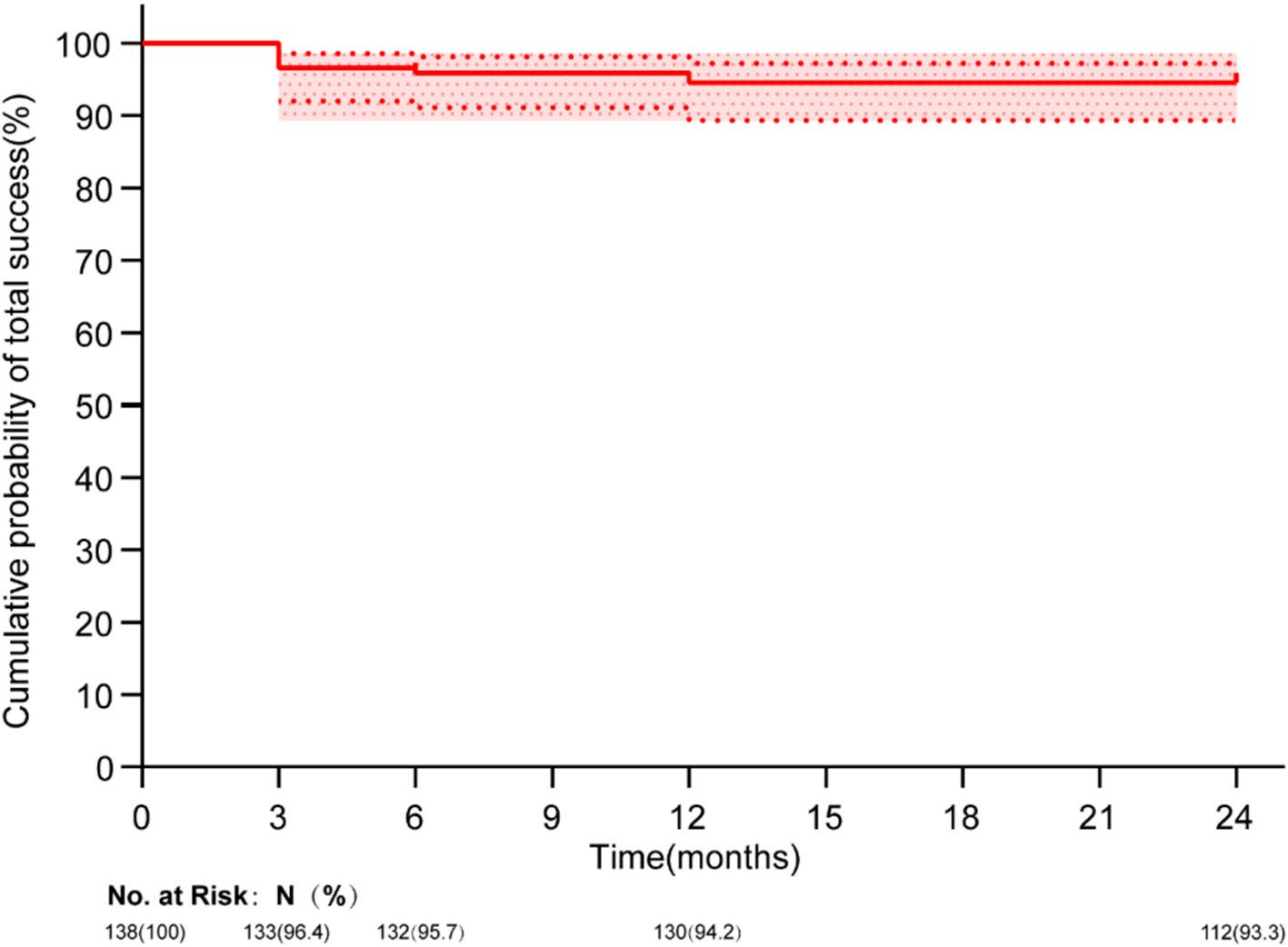
Kaplan–Meier survival plots of the cumulative probabilities of total success of OAG eyes.

The total success rate of surgery was 93.3%, the complete success rate, 82.5% and the conditional success rate 10.8% at 2 years (Table 4). The Kaplan–Meier survival analysis of the cumulative probabilities of total success of OAG eyes is exhibited in Figure 5. The most notable complication was hyphema, which occurred in 79.0% (109/138) eyes (Table 5). Hyphema was absorbed spontaneously in 88 eyes within 1 week, while the remaining 21 eyes recovered within 4 weeks without surgical intervention. IOP was elevated in 23 eyes (16.7%) transiently within 1 month after surgery and was reduced after topical use of glaucoma medications (Table 5). Descemet’s membrane detachment occurred in nine eyes (6.5%), and ciliary body detachment in four eyes (2.9%) (Table 5). No severe complications, such as choroidal detachment, iris tear, shallow anterior chamber, malignant glaucoma, vitreous hemorrhage, or persistent hypotony, were recorded.

**Table 4:** Rates of total, complete, and conditional success.

Follow-up time	3 months	6 months	1 year	2 years
*N*	138	138	138	120
Total success, *N* (%)	133 (96.4%)	132 (95.7%)	130 (94.2%)	112 (93.3%)
Complete success, *N* (%)	130 (94.2%)	128 (92.8%)	117 (84.8%)	99 (82.5%)
Conditional success, *N* (%)	3 (2.2%)	4 (2.9%)	13 (9.4%)	13 (10.8%)

**Table 5:** Postoperative complications in 138 OAG eyes.

Complications	OAG (*n* = 138)
Hyphema	109 (79.0%)
Transient IOP elevation (>21 mmHg)	23 (16.7%)
21 mmHg < IOP ≤ 25 mmHg	7 (5.1%)
25 mmHg < IOP ≤ 30 mmHg	6 (4.3%)
IOP > 30 mmHg	10 (7.2%)
Descemet’s membrane detachment	9 (6.5%)
Ciliary body detachment	4 (2.9%)

## 4 Discussion

Circumferential trabeculotomy, a type of SC surgery, has proven highly successful in both adult and pediatric patients with glaucoma (17, 18). In the present study, we first reported that our modified external suture trabeculotomy achieved a total success rate of 93.3% at 2 post-operative years (Table 4). Notably, the complete success rates were 84.8% at 1 year and 82.5% at 2 years in our study, significantly higher than those mentioned in one previous study (2). The current study showed the feasibility and favorable outcomes of this novel surgical procedure. As shown in our study, the following modifications may simplify the surgery and shorten operating time. First, we only made an L-shaped scleral flap (Figure 1c). We neither prepared nor removed a deep scleral flap, nor sutured the conjunctival incision as mentioned in most of the current studies (2). Second, in cross-section, SCs are elongated ellipses, with a longer axis of 150–350 μm (19). The diameter of the 6–0 suture is 70 μm, while the i-track is a beacon-tipped, 250-μm-thick microcatheter connected to an ophthalmic visco surgical device (20). Due to the thinness of the suture, we only needed to make a small incision under the superficial scleral flap to open the outer wall of SC.

Previous studies have shown that the incidence of PAS after trabeculotomy in patients with OAG is as high as 86% (14). We speculated that the inclusion of an iridectomy might help to balance the pressure between the anterior and posterior chambers in the early postoperative period and reduce the occurrence of postoperative PAS, thus improving the clinical outcome of external suture trabeculotomy. In this study, we designed iridectomy at the root in the modified external suture trabeculotomy and reported a high overall success rate of 93.3%, with a proportion of patients (82.5%) still showing a medication-free control of IOP at 2 years. However, we did not record the incidence and extent of post-operative PAS in patients in this study. More randomized controlled trials should be conducted to compare the degrees of post-operative PAS in patients with OAG who have undergone trabeculotomy with or without iridectomy.

Notably, the overall success rate of catheterization with our modified method is 98.6% (Table 2). Precise SC localization, a key procedure of our novel technique, is accomplished as follows: a superficial scleral flap, 4 × 4 mm in size and 1/2 of scleral thickness, was forwarded into the clear corneal limbus by approximately 1 mm. Then, the clear corneal and white scleral areas were identified. The gray zone, also known as the grayish blue trabecular meshwork, was delineated. At the posterior half of this zone, the SC was accurately located (16) (Figure 1d).

When performing this novel surgical method, caution should be paid to the following aspects. At the beginning of catheterization, forceps are used to lift the upper wall of the SC and the surrounding tissues to better expose the SC (Figures 1d–f). Next, the 6–0 suture is advanced circumferentially by 360 degrees into the canal (Figures 1g,h). In some cases, the suture may be resisted and misdirected, and the force cannot be further transmitted forward to the bend of the SC. Then the conjunctiva is incised, and a scleral flap is cut downward at the point of the presumed obstruction. After pulling out the suture locally, “relay catheterization” is performed (Figure 3). It is easier to impose force as the end of the suture is closer to the part held by the forceps. If the cannulation still cannot be performed smoothly, a 5–0 polypropylene suture is used instead. If false passages frequently occur during cannulation, a 7–0 polypropylene suture is recommended.

There are some limitations in this study. First, only OAG patients in China were included, and our surgery may not be suitable for OAG patients in other countries. Second, the inclusion criteria in our research required successful modified external suture trabeculotomy and a follow-up of at least 24 months. This may exclude patients with poor follow-up, complications, or early failures, thereby overestimating success rates. Third, more randomized controlled trials should be conducted in the near future to directly compare the efficacy, safety, and cost-effectiveness of this procedure with traditional surgical methods.

## 5 Conclusion

Simple and cost-saving, our modified external suture trabeculotomy provides favorable 2-year outcomes in treating medically uncontrolled OAG, as shown by short surgery duration, effective IOP control, obvious drug reduction, and less post-operative interventions.

## Data Availability

The original contributions presented in the study are included in the article/supplementary material, further inquiries can be directed to the corresponding authors.
